# Strontium-Modified BaTiO_3_ Composite Nanogenerators for Flexible and Self-Powered Human–Machine Interactions

**DOI:** 10.1021/acsomega.6c01611

**Published:** 2026-08-10

**Authors:** Bojja Naveen Kumar, Kaliyannan Manojkumar, Thamil Selvi Velayutham, Suman Maloji, G. R. K. Prasad, Bhaskar Dudem, Venkateswaran Vivekananthan

**Affiliations:** † Centre of Excellence in Flexible Electronics, Department of Electronics and Communication Engineering, 207673 Koneru Lakshmaiah Education Foundation, Guntur 522502, Andhra Pradesh, India; ‡ Low Dimensional Materials Research Centre, Department of Physics, Faculty of Science, Universiti Malaya, Kuala Lumpur 50603, Malaysia; § Department of Integrated Research and Discovery, 207673 Koneru Lakshmaiah Education Foundation, Guntur 522502, Andhra Pradesh, India; ∥ Advanced Technology Institute, Department of Electrical and Electronic Engineering, 3660 University of Surrey, Guildford GU2 7XH, England, United Kingdom; a Z-PULSE Ltd., New Cambridge House, Bassingbourn Road, Royston, Cambridgeshire, SG8 0SS, UK

## Abstract

Flexible piezoelectric nanogenerators (PENGs) have emerged as promising energy-harvesting devices for wearable electronics and self-powered human–machine interfaces. In this study, barium strontium titanate (BSRT) with varying Sr-substitution ratios was synthesized via a conventional solid-state reaction route, and its structural and functional properties were systematically evaluated. X-ray diffraction analysis confirmed the formation of phase-pure tetragonal perovskite BSRT and successful incorporation of Sr into the BaTiO_3_ lattice. The composition with 5% Sr exhibited optimized structural characteristics and was selected for device fabrication. Flexible BSRT-polydimethylsiloxane (PDMS) composite films were prepared and employed as the active layer in a sandwich-structured PENG. The electrical output performance was tuned by varying the filler loading, with an optimal composition of 15 wt % yielding a peak-to-peak voltage of 60 V and a short-circuit current of 5 μA under periodic mechanical excitation. The device further demonstrated stable capacitor charging behavior, excellent mechanical durability, and the capability to power multiple light-emitting diodes. Additionally, a self-powered human–machine interface was demonstrated by integrating multiple PENG units with a microcontroller, enabling real-time slide navigation through simple finger tapping motions. This work highlights the potential of BSRT-based flexible PENGs as efficient energy harvesters and self-powered sensing platforms for next-generation wearable and interactive electronic systems.

## Graphical abstract

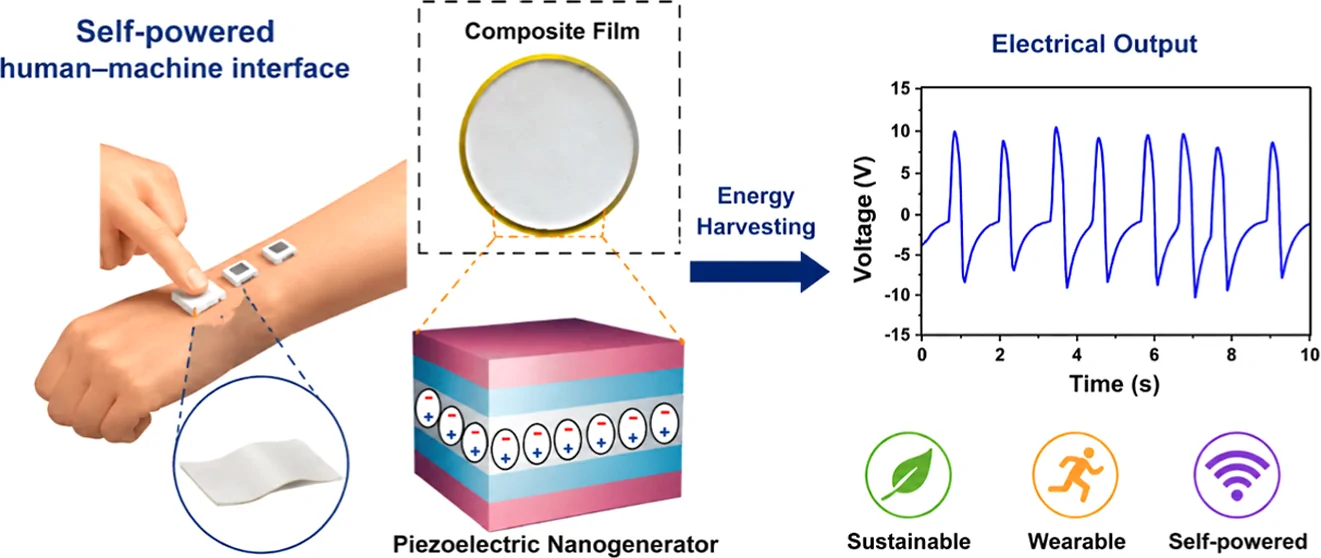

## 1 Introduction

The rapid development of wearable electronics,^1–3^ soft robotics,^4^ and human–machine interfaces has intensified the demand for sustainable,^5^ lightweight,^6,7^ and flexible power sources capable of operating without external batteries.^8–10^ Conventional energy storage systems suffer from limited lifetime, frequent recharging requirements, and environmental concerns, which significantly restrict their long-term applicability in wearable and autonomous systems^11–18^ In this context, mechanical energy-harvesting technologies have emerged as promising alternatives for enabling self-powered electronic devices by converting ambient biomechanical motions into useable electrical energy.^19–21^ Among various energy-harvesting mechanisms, piezoelectric nanogenerators (PENGs) have gained significant attention due to their high energy conversion efficiency, simple device architecture, and capability to operate under low-frequency mechanical excitations commonly generated by human motion.^22–25^ Piezoelectric ceramics such as lead zirconate titanate (PZT) exhibit excellent piezoelectric properties, however, their inherent brittleness, toxicity, and poor flexibility limit their applicability in wearable and environmentally friendly systems.^26,27^ As a result, lead-free perovskite-based materials have been extensively explored as alternative piezoelectric candidates.^28–34^ Barium titanate (BaTiO_3_) is one of the most widely studied lead-free piezoelectric materials owing to its high dielectric constant, strong ferroelectricity, and chemical stability.^35–37^ Pristine BaTiO_3_ often suffers from limited piezoelectric performance and structural instability under mechanical deformation.^38,39^ Despite substantial progress in BaTiO_3_-based piezoelectric nanogenerators, several key challenges continue to limit their practical implementation in flexible energy-harvesting systems. In particular, the optimal level of Sr-substitution required to achieve enhanced piezoelectric performance in BSRT remains insufficiently understood, and the correlation between compositional tuning, microstructure, and device output has not been clearly established. Moreover, the performance of BSRT–polymer composites is highly dependent on filler loading and dispersion, yet systematic optimization of these parameters is often lacking. In addition, the integration of flexible PENGs into functional, self-powered human–machine interface systems has received relatively limited attention. Elemental substitution at the A-site of the perovskite lattice has proven to be an effective strategy for tailoring the structural, dielectric, and piezoelectric properties of BaTiO_3_-based materials. In particular, partial substitution of Ba^2+^ with Sr^2+^ to form BSRT enables precise control over lattice distortion, phase stability, and polarization behavior, thereby offering enhanced functional performance.^40–42^ To realize flexible and wearable PENG devices, rigid ceramic piezoelectric materials must be integrated with soft polymer matrices. Polydimethylsiloxane (PDMS) is a widely used elastomer due to its excellent flexibility, biocompatibility, mechanical robustness, and ease of processing.^43,44^ Incorporating piezoelectric ceramic fillers into PDMS matrices allows the fabrication of flexible composite films that effectively combine mechanical compliance with functional piezoelectric activity.^45–47^ However, the electrical output performance of such composites strongly depends on ceramic composition, filler loading, dispersion quality, and interfacial interactions, necessitating systematic optimization. In addition to energy harvesting, self-powered sensing and control systems based on PENGs have opened new opportunities for human–machine interfaces (HMIs). By directly converting mechanical stimuli into electrical signals without external power sources, PENG-based HMIs offer simplified architectures, enhanced portability, and improved energy efficiency compared to conventional input devices.^17,48–50^

In this work, BSRT with varying Sr-substitution levels was synthesized via a conventional solid-state reaction method, and its structural and microstructural properties were systematically investigated. Flexible BSRT-PDMS composite films were subsequently fabricated and utilized as the active layer in a sandwich-structured PENG. The electrical output performance of the device was optimized by systematically varying the composite filler loading, while the energy-harvesting capability was evaluated through capacitor charging measurements and practical demonstrations. Furthermore, a self-powered human–machine interface was developed by integrating multiple BSRT-based PENGs onto a wearable platform, enabling real-time slide navigation through finger tapping motions. These results demonstrate the practical applicability of the proposed system for wearable energy-harvesting and interactive control technologies.

## 2 Materials and Methods

### 2.1 Synthesis of BSRT

BSRT powders with different Sr-substitution ratios were synthesized using a conventional solid-state reaction method. High-purity barium carbonate (BaCO_3_), strontium carbonate (SrCO_3_), and titanium dioxide (TiO_2_) were used as precursor materials without further purification. Stoichiometric amounts of the precursors corresponding to Ba_1–*x*_Sr_*x*_TiO_3_ compositions (*x* = 0.01, 0.03, 0.05, 0.07, and 0.10) were accurately weighed and thoroughly mixed using an agate mortar and pestle for 30 min to ensure compositional homogeneity. The mixed powders were then subjected to calcination in a programmable muffle furnace. The temperature was increased from room temperature to 1100 °C at a controlled heating rate of 5 °C min^–1^ and maintained at this temperature for 4 h to promote solid-state diffusion and phase formation. The calcination was carried out under ambient air atmosphere. After the dwell time, the furnace was allowed to cool naturally to room temperature. The calcined powders were subsequently reground to break up agglomerates and improve particle uniformity. To further enhance phase purity and crystallinity, an optional second calcination step can be performed under identical conditions. The final BSRT powders were used for structural, morphological, and electrical characterization, as well as for the fabrication of flexible composite films.

### 2.2 BST Film Fabrication

Flexible BSRT-PDMS composite films were fabricated using a solution-free mechanical mixing approach. The PDMS elastomer base and curing agent (Sylgard 184) were first mixed in a fixed weight ratio of 10:1, following the manufacturer’s recommendation, and stirred for 10 min to obtain a homogeneous polymer matrix. Subsequently, BSRT powder (5% Sr composition) was added to the PDMS mixture at different weight fractions of 5, 10, 15, 20, and 25 wt % relative to the total composite mass. The mixture was mechanically stirred for 30 min to ensure uniform dispersion of the ceramic particles within the polymer matrix. The resulting composite slurry was then degassed under vacuum for 15–20 min to remove trapped air bubbles. After degassing, the mixture was cast into Petri dishes to form films with a thickness of approximately 0.5–1 mm. The films were thermally cured in an oven at 60 °C for 4 h under ambient atmosphere to achieve complete cross-linking of the PDMS network. After curing, the flexible composite films were carefully peeled off and cut into desired dimensions. For device fabrication, aluminum (Al) foil electrodes were attached to both sides of the composite film to form a sandwich-structured PENG. Finally, a thin PDMS encapsulation layer was applied to improve mechanical stability and electrical insulation.

### 2.3 Electrical Output Measurement and Testing Conditions

The electrical output performance of the fabricated BSRT-based piezoelectric nanogenerators was evaluated under controlled periodic mechanical excitation using a programmable linear motor. A vertical compressive force of 5 N was applied to the device at a fixed frequency of 2 Hz, generating a repeated contact-separation motion. The output voltage signals were recorded using a digital oscilloscope, while the short-circuit current was measured using a high-sensitivity electrometer (Keithley 6514). All measurements were conducted under ambient laboratory conditions (room temperature and atmospheric pressure). The effective contact area of the device during testing was approximately 3 × 3 cm^2^. Each device was subjected to multiple loading–unloading cycles to ensure measurement consistency, and the reported output values represent stable and reproducible performance under identical experimental conditions.

## 3 Results and Discussion

### 3.1 Structural Design, Phase Formation, and Spectroscopic Analysis of BSRT

The conceptual design and practical demonstration of a smart self-powered wristband incorporating a flexible BSRT-based piezoelectric nanogenerator is studied. The device is ergonomically designed to conform to the human wrist, enabling efficient collection of biomechanical energy generated from natural hand and wrist movements during daily activities. The flexible structure ensures mechanical stability and user comfort, which are essential for long-term wearable applications. The integrated piezoelectric sensing units convert externally applied mechanical deformation into electrical signals without the need for an external power source. This self-powered configuration highlights the suitability of the BSRT-based PENG for wearable sensing and human–machine interface applications. The wristband architecture demonstrates the practical potential of flexible piezoelectric devices for real-time motion monitoring, gesture recognition, and next-generation self-sustained wearable electronics in Figure 1a. A schematic illustration of the device configuration showed the layer-by-layer assembly of the flexible BSRT-based nanogenerator. The device consists of a BSRT-PDMS composite film sandwiched between aluminum (Al) electrodes, with additional PDMS encapsulation layers to enhance structural integrity and electrical insulation. This sandwich-type architecture ensures efficient charge collection while preserving flexibility and mechanical durability. BSRT powders were synthesized using a conventional solid-state reaction technique, 1–10% stoichiometric amounts of BaCO_3_, SrCO_3_, and TiO_2_ precursor materials were thoroughly mixed and subsequently calcined at 1100 °C for 4 h to obtain the desired perovskite phase (Figure 1b and c). A schematic illustration of the synthesis route, including precursor mixing, calcination, and final product formation, is presented in Figure 1d–f, while the detailed synthesis procedure is described in the Materials and Characterization section. The high-temperature calcination facilitates solid-state diffusion and promotes the formation of a crystalline BSRT structure. X-ray diffraction (XRD) patterns of BSRT samples with different Ba/Sr composition ratios of 1, 3, 5, 7, and 10% were obtained. The diffraction data were collected over a 2θ range of 20–90°, ensuring comprehensive phase identification and structural analysis. All diffraction peaks are sharp and well-defined, indicating high crystallinity of the synthesized materials. The observed diffraction peaks can be indexed to a tetragonal perovskite structure and match well with the standard reference pattern of BaTiO_3_-based perovskites (JCPDS no. 05-0626), confirming the successful formation of phase-pure BSRT without detectable secondary phases. The prominent diffraction peaks located at approximately 2θ = 22.2°, 31.5°, 38.9°, 45.3°, 50.8°, 56.2°, and 65.9° correspond to the (100), (101), (110), (111), (200), (211), and (220) crystallographic planes, respectively. The absence of impurity-related peaks associated with BaCO_3_, SrCO_3_, or TiO_2_ further verifies the complete solid-state reaction and high phase purity of the BSRT samples. With increasing Sr content, a slight systematic shift of diffraction peaks toward higher 2θ values is observed, which can be attributed to lattice contraction caused by the partial substitution of larger Ba^2+^ ions (ionic radius 1.61 Å) with smaller Sr^2+^ ions (ionic radius 1.44 Å). This confirms the formation of a solid-solution structure and effective incorporation of Sr into the BaTiO_3_ lattice. The lattice parameters were estimated from the indexed diffraction peaks, revealing a gradual decrease in lattice constants with increasing Sr concentration, consistent with Vegard’s law. This lattice distortion reflects compositional tuning within the perovskite framework and plays a crucial role in modifying the structural and functional properties of BSRT. Among all compositions, the BSRT sample with a 5% Sr ratio exhibits the most intense and narrow diffraction peaks, indicating improved crystallinity and reduced lattice strain compared to other compositions.

**Figure 1:**
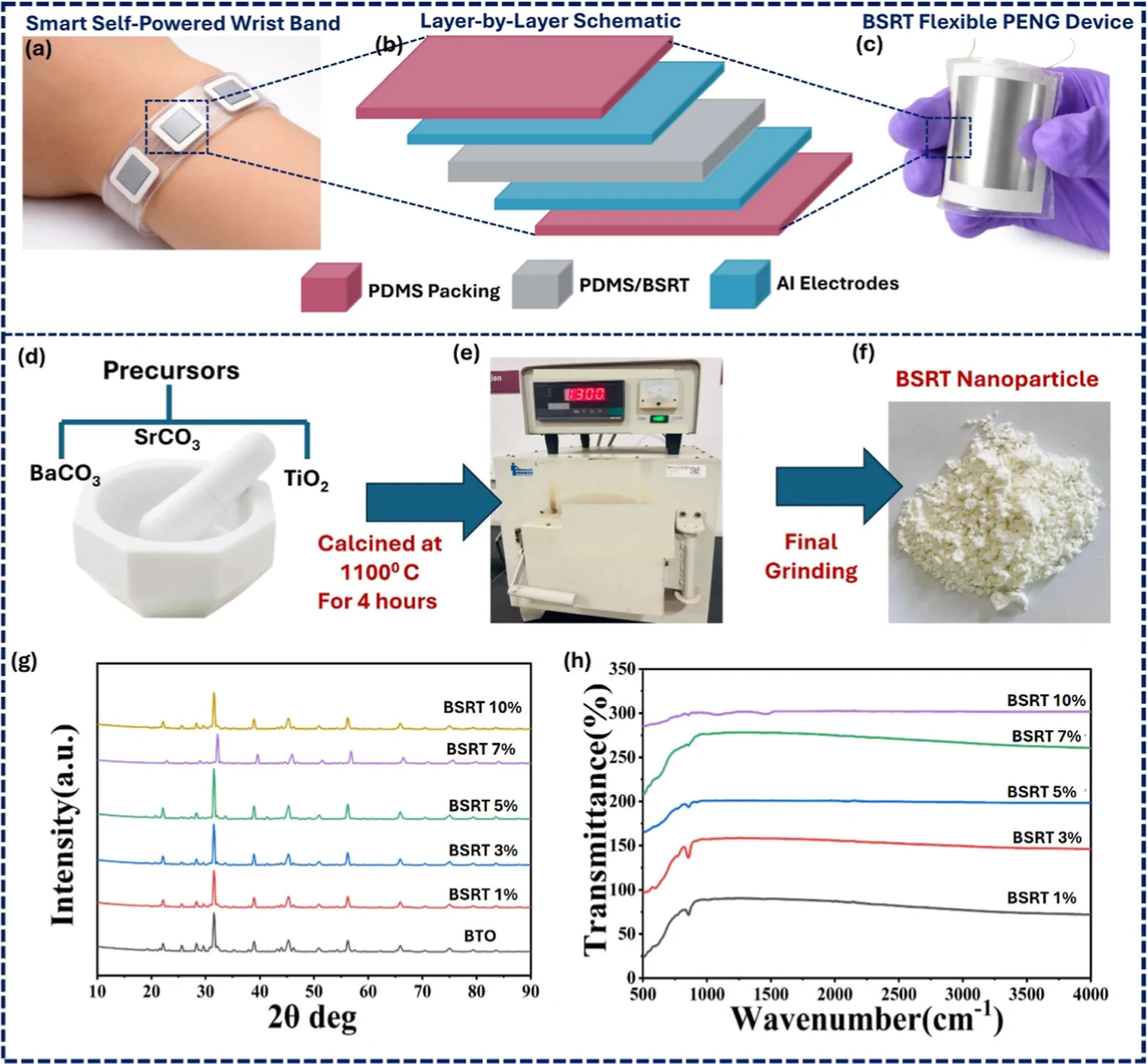
(a) Conceptual design and photographic demonstration of a flexible BSRT-based PENG integrated into a wristband, designed to harvest biomechanical energy from natural wrist motions. (b and c) Schematic of the device architecture showing a BSRT-PDMS composite active layer sandwiched between Al electrodes and encapsulated with PDMS for flexibility and durability. (d–f) Illustration of the solid-state synthesis process involving mixing of BaCO_3_, SrCO_3_, and TiO_2_ precursors followed by calcination to form perovskite BSRT. (g) XRD patterns of BSRT with varying Sr content (1–10%) confirming a tetragonal perovskite phase without impurities, with peak shifts toward higher 2θ indicating lattice contraction due to Ba^2+^/Sr^2+^ substitution and highest crystallinity at 5% Sr. (h) FTIR spectra showing characteristic Ti–O stretching (500–600 cm^–1^) and Ba–O/Sr–O lattice vibrations (650–750 cm^–1^), confirming phase purity and solid-solution formation. Overall, the structural and spectroscopic analyses verify successful synthesis and effective Sr incorporation in BSRT materials.

The average crystallite size (D) of the BSRT samples was estimated using the Scherrer equation

1 D=Kλ/βcos⁡θ

where *K* is the shape factor (0.9), λ is the X-ray wavelength (Cu Kα, 1.5406 Å), β is the full width at half-maximum (fwhm) of the diffraction peak, and θ is the Bragg angle. The calculated crystallite sizes fall within the nanometer range and show an increasing trend up to the 5% Sr composition, followed by a slight decrease at higher Sr content. This behavior suggests that moderate Sr incorporation promotes crystallite growth and structural ordering, whereas excessive substitution induces lattice strain that limits crystallite size. The XRD analysis confirms that the solid-state synthesis route enables the formation of phase-pure, highly crystalline BSRT materials with tunable structural parameters. The optimized crystallinity and lattice stability observed at the 5% Sr composition make it a promising candidate for enhanced dielectric and functional performance (Figure 1g). In the low-wavenumber region (<1000 cm^–1^), the FTIR spectra of BSRT nanoparticles exhibit distinct absorption bands characteristic of metal–oxygen lattice vibrations within the perovskite structure. The strong absorption band observed in the range of 500–600 cm^–1^ is attributed to the Ti–O stretching vibration of the TiO_6_ octahedra, which is a fingerprint feature of perovskite titanates. Additional bands appearing in the region of 650–750 cm^–1^ are assigned to Ba–O and Sr–O lattice vibrations, confirming the occupancy of Ba^2+^ and Sr^2+^ ions at the A-site of the perovskite lattice. The presence and consistency of these characteristic absorption bands across all compositions indicate the formation of a stable BSRT solid solution without detectable secondary or impurity phases. With increasing Sr content, a gradual change in the transmittance intensity and slight band broadening are observed in the metal–oxygen vibration region. These variations are attributed to local lattice distortion and changes in bond strength arising from the substitution of larger Ba^2+^ ions by smaller Sr^2+^ ions at the A-site, which alters the Ti–O bond environment and lattice dynamics. The systematic evolution of the Ti–O and A-site metal–oxygen vibration bands with Sr concentration confirms the effective incorporation of Sr^2+^ ions into the BaTiO_3_ lattice. Such lattice modifications are known to influence polarization behavior by affecting octahedral tilting and domain dynamics, which are directly related to dielectric and piezoelectric properties in Figure 1h. FTIR analysis verifies the chemical integrity, compositional uniformity, and phase stability of the BSRT nanoparticles, supporting their suitability for flexible piezoelectric nanogenerator applications.

### 3.2 Morphological and Surface Topographical Analysis of BSRT

The scanning electron microscopy (SEM) image of the synthesized BSRT powder is analyzed. The micrograph reveals irregularly shaped particles with noticeable agglomeration, which is typical of solid-state-derived composite materials. The particle size is predominantly in the micro meter range, and the relatively rough surface morphology suggests grain growth during the high-temperature calcination process. Such microstructural features are beneficial for enhancing interfacial interactions and dielectric behavior in perovskite-based materials in Figure 2a. The elemental composition of BSRT was further analyzed using energy-dispersive X-ray spectroscopy (EDS). The EDS spectrum confirms the presence of Ba, Sr, Ti, and O elements without detectable impurity-related peaks, indicating the successful incorporation of Sr into the barium titanate lattice and the absence of secondary phases. The elemental distribution verifies the compositional homogeneity of the synthesized BSRT material, as shown in Figure 2b. Atomic force microscopy (AFM) was employed to investigate the nanoscale surface topography of the BST film. The two-dimensional height image over a scan area of 5 × 5 μm^2^ reveals a relatively uniform surface with fine granular features. The corresponding height distribution and histogram indicate limited height variation across the scanned region, reflecting a smooth and homogeneous film surface. Such surface uniformity is favorable for reducing electrical leakage and enhancing electromechanical coupling displayed in Figure 2c.

**Figure 2:**
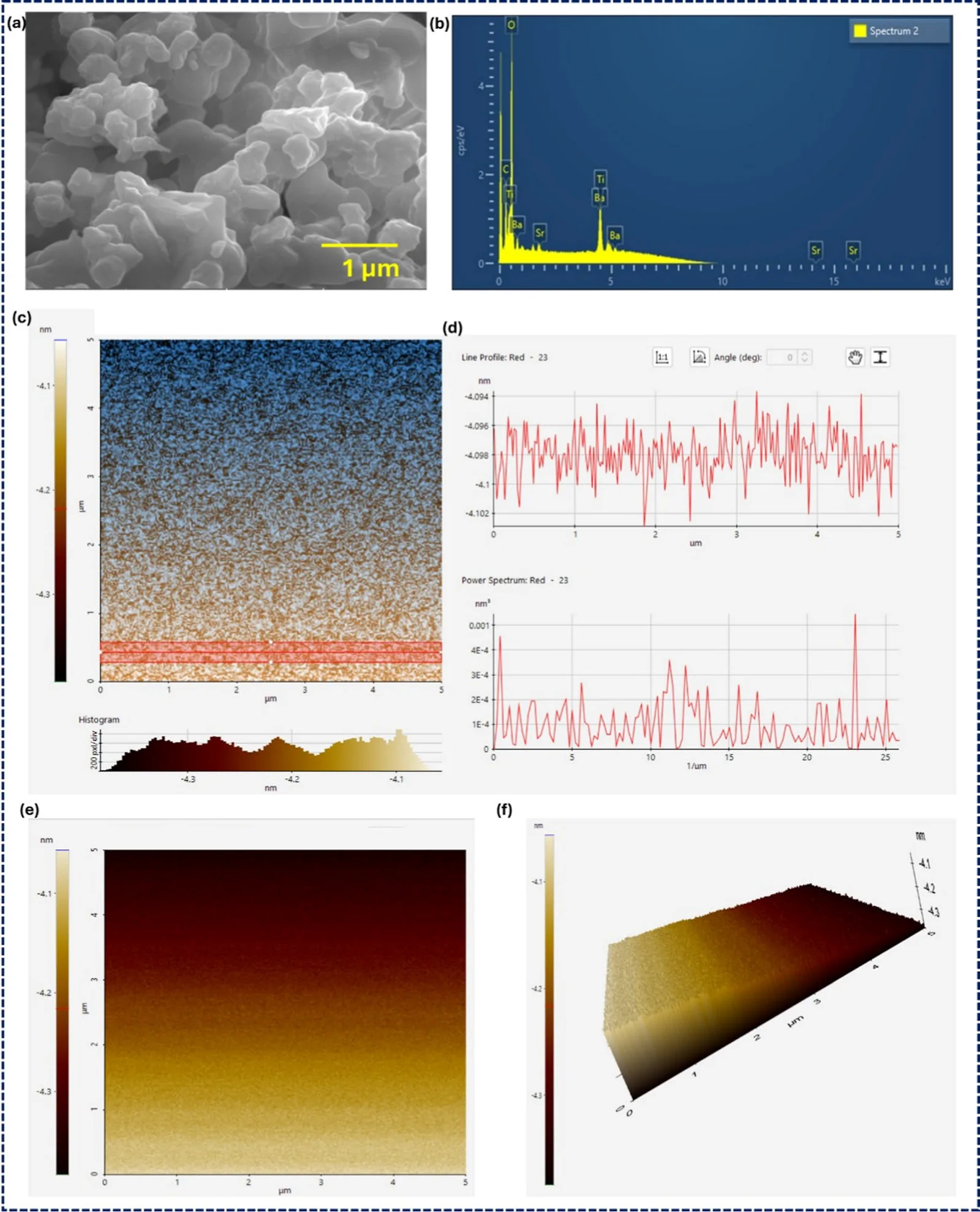
(a) SEM image of synthesized BSRT powder showing irregular, agglomerated particles with micro meter-scale sizes and rough surfaces due to grain growth during high-temperature calcination. (b) EDS spectrum confirming the presence of Ba, Sr, Ti, and O elements with no detectable impurities, indicating successful Sr incorporation and compositional uniformity. (c) AFM height image (5 × 5 μm^2^ scan area) displaying a uniform surface with fine granular morphology and minimal height variation. (d) AFM line profile with corresponding power spectral density (PSD) analysis, revealing controlled nanoscale roughness dominated by short-range spatial features. (e) AFM error signal image highlighting grain boundaries and subtle surface features not clearly visible in the height image. (f) Three-dimensional AFM surface reconstruction showing smooth morphology with gradual height variations across the BSRT film.

To further quantify the surface characteristics, AFM line profile analysis and power spectral density plots were extracted from the selected region. The line profile demonstrates nanoscale height fluctuations, confirming controlled surface roughness, while the power spectrum suggests the dominance of short-range spatial features. These characteristics are indicative of well-distributed surface grains and minimal long-wavelength roughness, as shown in Figure 2d. The AFM error signal image provides an enhanced contrast of fine surface features, highlighting grain boundaries and subtle height variations that are not easily discernible in conventional height images in Figure 2e. Additionally, the three-dimensional AFM surface reconstruction offers a comprehensive visualization of the surface morphology, clearly illustrating the gradual height variations and overall smoothness of the BST film in Figure 2f.

### 3.3 Fabrication Process and Mechanical Flexibility of BSRT–PDMS Composite Films

The step-by-step fabrication process of the flexible BSRT-PDMS composite film is studied. The process begins with the preparation of the PDMS matrix by mixing the base elastomer with the curing agent, followed by the incorporation of BSRT composite into the PDMS to ensure a uniform and homogeneous dispersion of the piezoelectric filler within the polymer matrix (Figure 3a and b). The resulting mixture is then cast into a Petri dish and thermally cured at 60 °C, allowing the PDMS chains to undergo cross-linking and form a mechanically stable composite film (Figure 3c). After curing, the composite film is carefully peeled off from the Petri dish to obtain a free-standing flexible BSRT-PDMS film, which serves as the core piezoelectric active layer for device fabrication. Digital photographic images demonstrate the free-standing film in Figure 3d. The excellent mechanical flexibility of the fabricated BSRT composite film is observed. Digital photographic images demonstrate that the free-standing film can be easily stretched, twisted, bent, and rolled without visible cracks or mechanical degradation, confirming its outstanding mechanical robustness and suitability for flexible and wearable electronic applications. Such mechanical resilience is essential for maintaining stable electrical performance under repeated deformation in Figure 3e.

**Figure 3:**
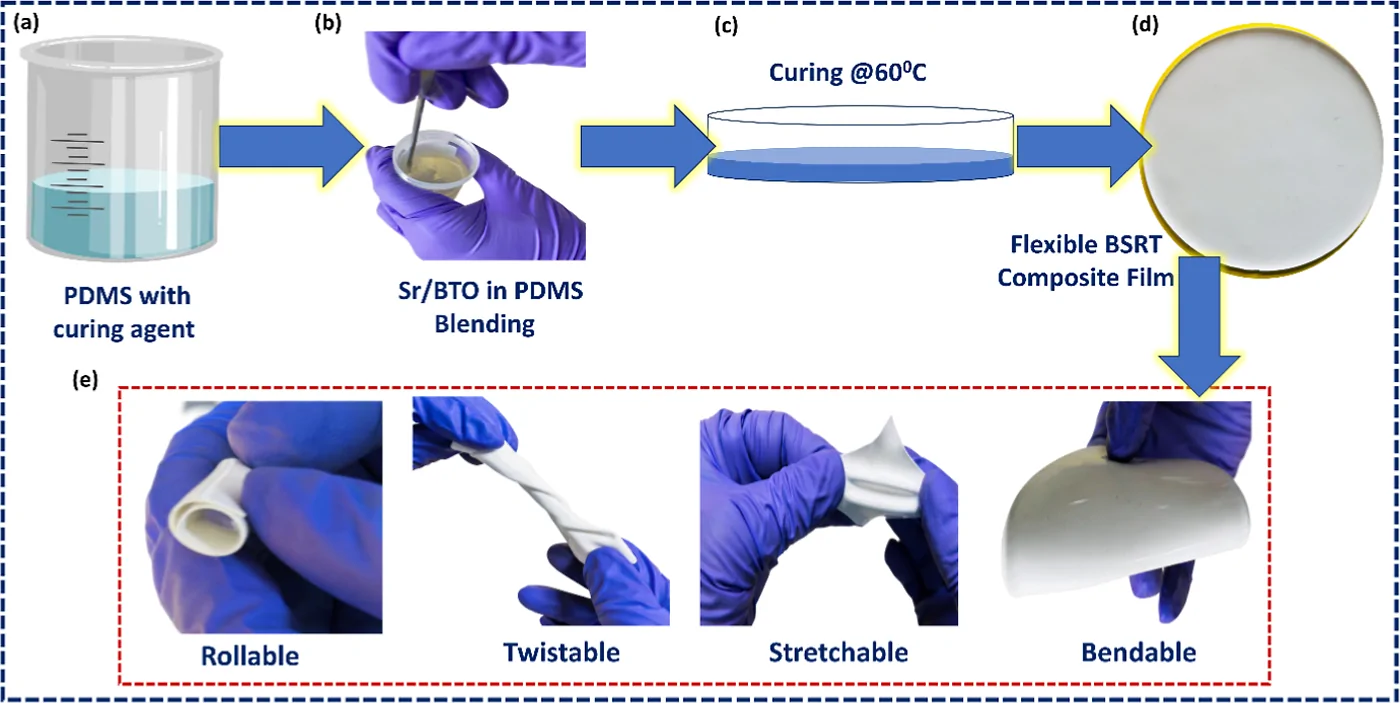
(a–c) Step-by-step schematic of the fabrication of the flexible BSRT-PDMS composite film, including mixing of PDMS base and curing agent, uniform dispersion of BSRT fillers, casting into a Petridis, and thermal curing at 60 °C to obtain a free-standing composite film. (d) Digital photograph of BSRT film (e) shows mechanical flexibility under stretching, twisting, bending, and rolling, demonstrating robustness and suitability for wearable applications.

### 3.4 Working Mechanism and Electrical Output Performance of the BSRT-Based PENGs

Mechanical deformation of the BSRT-PDMS composite induces charge separation and polarization within the piezoelectric layer, resulting in the generation of an alternating electrical output during repeated loading and unloading cycles. The piezoelectric energy-harvesting process operates through three distinct stages (Figure 4a­(i)) in the initial state, when no external force is applied, the dipoles within the BSRT composite layer remain randomly oriented, resulting in no net polarization or electrical output (Figure 4a­(ii)) upon mechanical pressing, external stress induces deformation of the BSRT lattice, leading to dipole alignment and the generation of an internal piezoelectric potential, which drives charge accumulation on the opposing electrodes and Figure 4a­(iii) during the release of the applied force, the polarization decreases and reverses, causing electrons to flow back through the external circuit and generating an alternating electrical signal. This cyclic deformation recovery process enables the flexible BSRT nanogenerator to effectively convert mechanical energy to electrical energy, making it a promising candidate for self-powered flexible electronics and wearable sensing systems.

**Figure 4:**
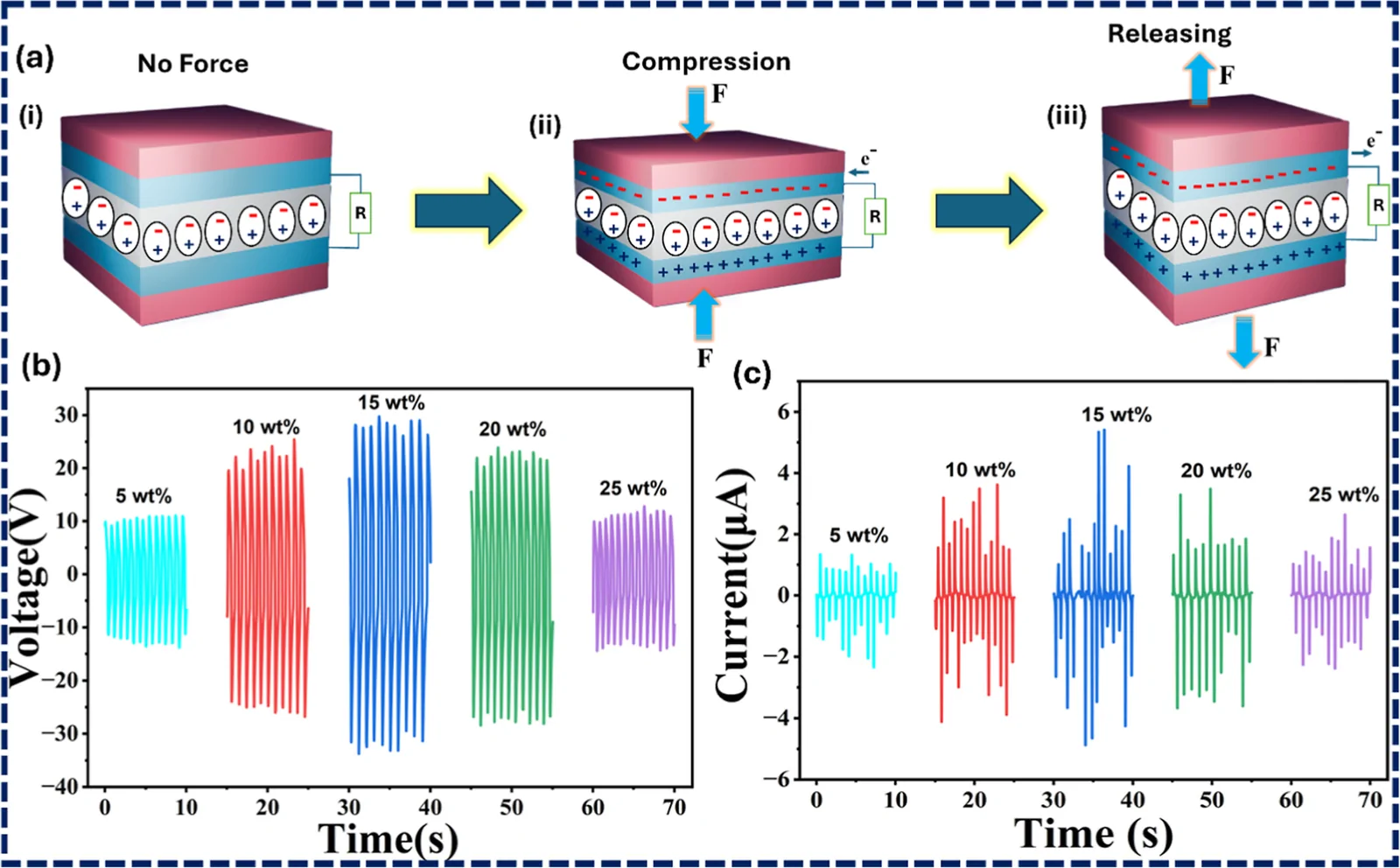
(a) (i, ii, and iii) Schematic illustration of the working mechanism of the BSRT-based piezoelectric nanogenerator, where mechanical stress induces dipole alignment and charge separation, generating an electrical potential. Upon release of stress, polarization reverses, leading to charge redistribution and current flow, producing an alternating electrical output. (b) Open-circuit voltage showing periodic peak-to-peak signals, increasing up to 60 V at 15 wt % due to enhanced stress transfer and polarization, followed by a decrease at higher ratios due to particle agglomeration. (c) Short-circuit current exhibiting alternating peaks, reaching 5 μA at 15 wt % due to improved charge transport, and decreasing at higher ratios due to increased resistance and reduced deformability.

The electrical output characteristics of the flexible BSRT-based piezoelectric nanogenerator, in which the BSRT filler content was fixed at 5% while five different composite ratios (5, 10, 15, 20, and 25 wt %), were systematically evaluated under identical mechanical excitation conditions. As the composite ratio increases from 5 to 15 wt %, the output voltage increases, reaching a maximum peak-to-peak voltage of approximately 60 V at 15 wt %. This improvement can be attributed to the increased effective piezoelectric active volume and enhanced stress transfer from the flexible PDMS matrix to the BSRT particles, which promotes stronger dipole polarization and higher piezoelectric potential generation. However, when the composite ratio is further increased to 20 and 25 wt %, the output voltage shows a gradual decline. This reduction is primarily associated with particle agglomeration at higher filler contents, which leads to nonuniform stress distribution, reduced interfacial bonding, and mechanical stiffening of the composite, thereby limiting effective dipole alignment and polarization under applied stress shown in Figure 4b. A similar trend is observed in the short-circuit current output. The current output increases steadily with increasing composite ratio up to 15 wt %, reaching a maximum value of approximately 5 μA. The enhanced current generation at this optimal ratio is attributed to the improved connectivity between BSRT particles and the formation of more efficient charge transport pathways within the composite matrix. Additionally, the higher polarization density at this composition facilitates faster charge separation and migration toward the electrodes. In contrast, at higher composite ratios (20 and 25 wt %), the current output decreases due to increased internal resistance, restricted polymer chain mobility, and reduced deformation capability of the composite. These factors collectively impede charge transport and suppress the effective piezoelectric response, as illustrated in Figure 4c.

### 3.5 Energy Storage Capability and Stability Performance of the BSRT-Based PENGs

The energy storage capability of the optimized BSRT-based PENGs was further investigated using external capacitors. The device demonstrates a stable charging behavior for different capacitance values (1 μF, 3.3 μF, and 4.7 μF), with smaller capacitors exhibiting faster voltage accumulation due to their lower charge storage requirements, as shown in Figure 5a. The monotonic voltage increase confirms the efficient conversion of mechanical energy into stored electrical energy. A magnified view of the voltage output under continuous mechanical excitation, revealing a consistent and stepwise voltage increment, indicates stable device operation and mechanical durability in Figure 5b. The cyclic charging and discharging characteristics further validate the reliability and repeatability of the BSRT-based PENGs. The well-defined voltage rise and decay cycles demonstrate efficient energy-harvesting and release processes under periodic mechanical stimulation, as shown in Figure 5c. Finally, the practical applicability of the device is demonstrated, where the harvested energy successfully powers multiple light-emitting diodes (LEDs) in Figure 5d. This result confirms that the optimized BSRT-based PENG can serve as an effective power source for low-power electronics and self-powered sensing systems. The cyclic test has been performed to evaluate the device stability, and the results demonstrate excellent cycle endurance with negligible degradation in Figure 5e.

**Figure 5:**
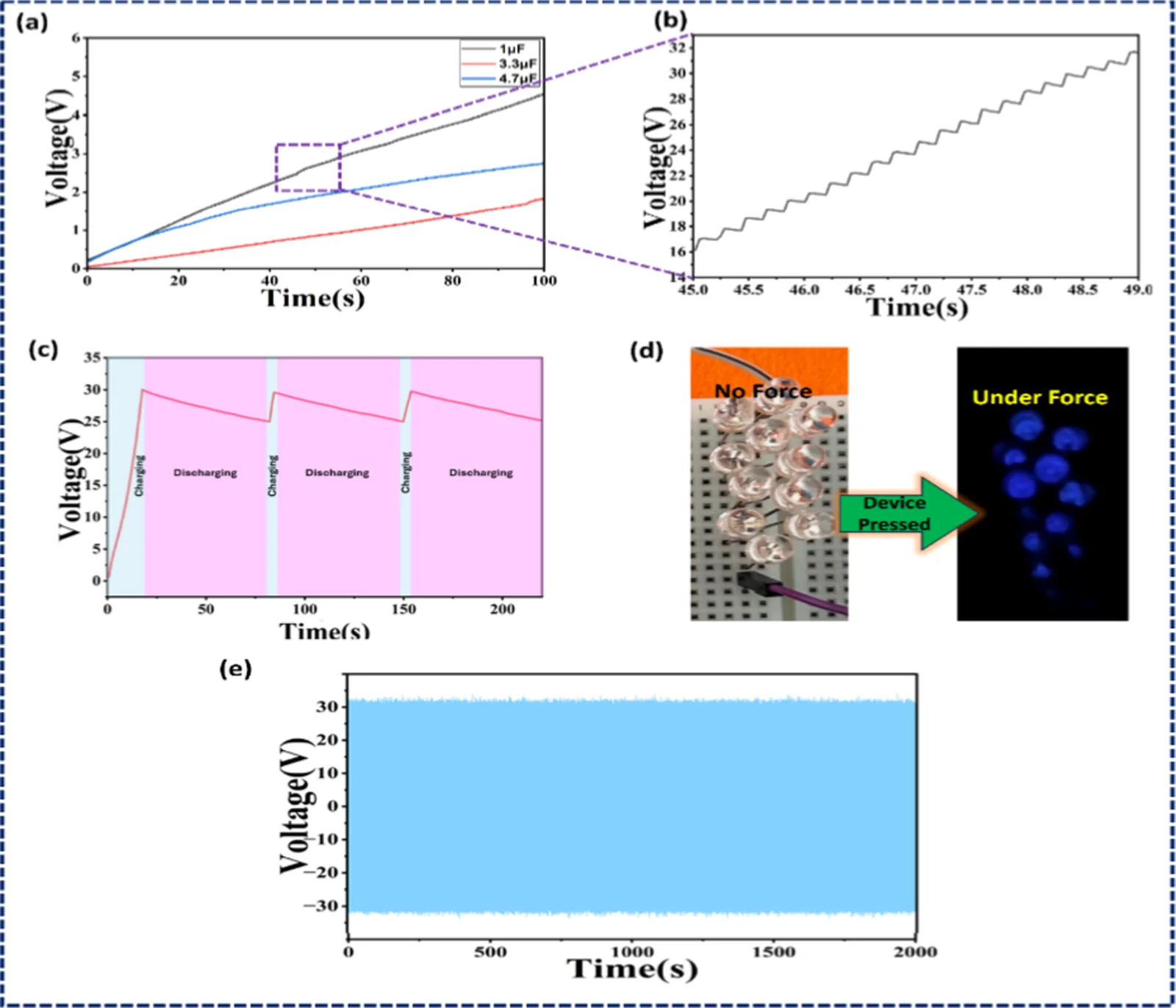
Electrical output and energy storage performance of the flexible BSRT-based PENGs. (a) Charging curves of capacitors (1, 3.3, and 4.7 μF), where smaller capacitors show faster voltage rise, confirming efficient energy storage. (b) Stepwise voltage increases (c) cyclic charging–discharging profiles indicating stable operation, durability, and repeatable energy conversion under continuous mechanical stimulation. (d) Demonstration of powering multiple LEDs, confirming the practical applicability of the optimized PENG for low-power electronic devices. (e) Long-term cycling tests have now been performed to evaluate the device stability, and the results demonstrate excellent cycle endurance with negligible degradation in output after repeated loading.

### 3.6 Self-Powered Human–Machine Interface Application of the BSRT-Based PENGs

A self-powered human–machine interface (HMI) application was enabled by the flexible BSRT-based piezoelectric nanogenerator. A schematic illustration of the working principle of the smart self-powered wristband system is shown. Mechanical stimuli generated by human motion are converted into electrical signals by the integrated BST piezoelectric film. The generated electrical output is subsequently subjected to signal extraction and conditioning, followed by signal processing using embedded algorithms. The processed signals are then translated into commands and delivered to the human–machine interface (HMI), enabling real-time interaction and communication without the need for an external power supply in Figure 6a. Three miniaturized PENG devices are integrated onto the user’s hand to harvest biomechanical energy generated during finger tapping motions. Each PENG functions as an independent self-powered sensor that converts mechanical tapping into electrical voltage signals without the need for an external power supply.

**Figure 6:**
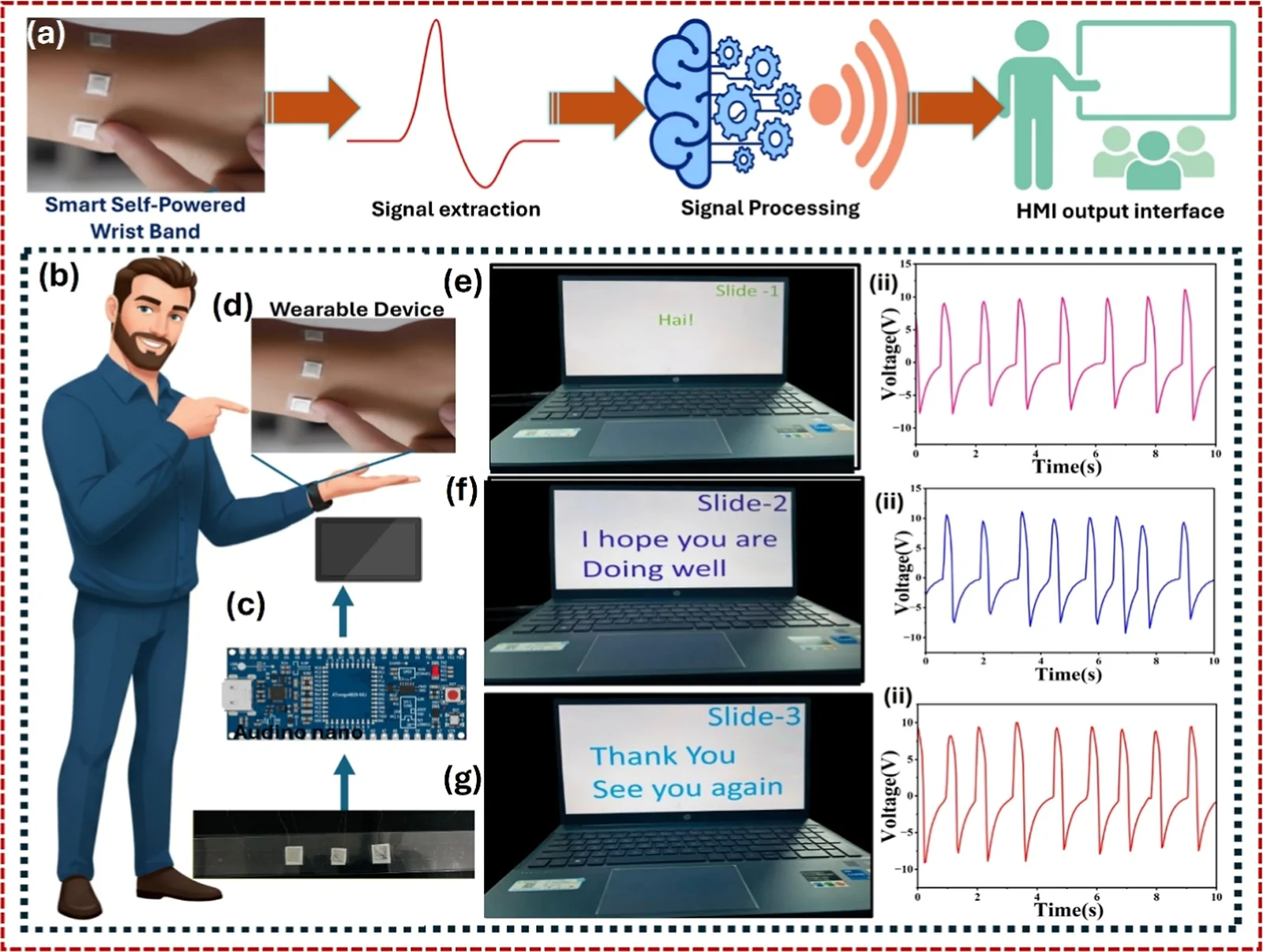
Self-powered human–machine interface (HMI) enabled by the flexible BSRT-based PENG. (a) Schematic illustration of the working principle of the smart self-powered wristband system, in which biomechanical motions are converted into electrical signals by the BSRT piezoelectric film, followed by signal conditioning, processing, and translation into HMI commands without external power. (b–d) Integration of three miniaturized BSRT-based PENG devices on the human hand for harvesting biomechanical energy during finger-tapping motions (b) system concept, (c) interfacing of the PENG outputs with an Arduino Nano microcontroller for signal detection and processing, and (d) photograph of the flexible PENG devices attached to the hand, demonstrating wearable applicability. (e–g) Output voltage waveforms generated by forward and reverse tapping of the three PENG sensors, where distinct voltage peaks are recognized and mapped to specific control commands for sequential slide navigation. Forward tapping enables slide advancement (slides 1–3), while reverse tapping induces opposite-polarity signals for backward navigation, demonstrating bidirectional, self-powered control. This demonstration confirms the feasibility of the BSRT-based PENG as a fully self-powered HMI platform for wearable electronics, gesture recognition, and interactive human–machine communication.

The generated electrical signals are transmitted to an Arduino Nano microcontroller, which serves as the signal processing and control unit in Figure 6b. The interfacing of the PENG devices with the Arduino Nano, where the output voltage pulses produced during tapping are detected, filtered, and converted into digital control signals, is shown in Figure 6c. The flexible BSRT-based PENG device is attached to the human hand, demonstrating its suitability for wearable and self-powered human–machine interface applications shown in Figure 6d. Upon forward tapping of the PENG devices, distinct voltage waveforms are generated, each tapping event produces a characteristic voltage peak that is recognized by the microcontroller and mapped to a specific command. Consequently, a single tap on the first PENG advances the display to Slide 1, tapping the second PENG switches the display to Slide 2, and tapping the third PENG activates Slide 3, demonstrating precise and sequential slide navigation from top to bottom. Tapping motions generate voltage signals with polarity characteristics, enabling bidirectional control. As illustrated in the corresponding voltage time plots, reverse tapping commands cause the system to navigate slides from bottom to top, allowing intuitive backward navigation without the need for additional input devices. This dual-direction control highlights the capability of the PENG to distinguish different mechanical stimuli based solely on the generated electrical signals. The successful demonstration of real-time slide control using hand-mounted PENG devices confirms the feasibility of this system as a fully self-powered HMI platform. By eliminating the requirement for external power sources and conventional input peripherals, this approach offers a lightweight, flexible, and energy-efficient solution for wearable electronics, smart presentation systems, and interactive human–machine communication. The proposed self-powered interface demonstrates significant potential for next-generation wearable control systems, gesture recognition, and assistive technologies, as illustrated in Figure 6e–g.

## 4 Conclusion

A flexible and efficient piezoelectric nanogenerator based on BSRT-PDMS composite films was successfully developed for energy harvesting and self-powered human–machine interface applications. Phase-pure BSRTs with a tetragonal perovskite structure were synthesized using a solid-state reaction method, and systematic Sr-substitution enabled effective tuning of lattice parameters and crystallinity. Among the investigated compositions, the BSRT sample with 5% Sr content exhibited optimized structural stability and crystallite growth, making it a suitable candidate for flexible piezoelectric devices. Free-standing BSRT-PDMS composite films with excellent mechanical flexibility were fabricated and integrated into a sandwich-type PENG architecture. The electrical output performance was strongly dependent on the composite filler loading, with an optimal composite ratio of 15 wt % yielding a maximum output voltage of approximately 60 V and a short-circuit current of about 5 μA. The optimized device demonstrated stable capacitor charging behavior, reliable cyclic performance, and sufficient power output to drive multiple LEDs, confirming its practical energy-harvesting capability. A self-powered human–machine interface was successfully demonstrated by integrating multiple BSRT-based PENG devices with a microcontroller to enable intuitive, bidirectional slide navigation through finger tapping motions. This demonstration highlights the ability of the proposed system to function simultaneously as an energy harvester and a self-powered sensor without the need for external power supplies. This work provides a viable strategy for designing high-performance, lead-free, and flexible piezoelectric nanogenerators and underscores their potential for wearable electronics, interactive control systems, and next-generation self-powered human–machine interfaces.

## Supplementary Material



## Notes

Wearable slide changer application demonstration (MP4)
